# Draft Genome Sequence of *Gordonia alkanivorans* Strain CGMCC6845, a Halotolerant Hydrocarbon-Degrading Bacterium

**DOI:** 10.1128/genomeA.01274-13

**Published:** 2014-01-30

**Authors:** Xinxin Wang, Decai Jin, Lisha Zhou, Liang Wu, Wei An, Lin Zhao

**Affiliations:** a School of Environmental Science and Engineering, Tianjin University, Tianjin, People’s Republic of China; b China Offshore Environmental Service Co., Ltd., Tianjin, People’s Republic of China; c College of Resources and Environment, University of Chinese Academy of Sciences, Beijing, People’s Republic of China; d Shenzhen Key Laboratory of Environmental Microbial Genomics and Application, BGI, Shenzhen, People’s Republic of China; e College of Environmental Science and Engineering, Ocean University of China, Qingdao, People’s Republic of China

## Abstract

*Gordonia alkanivorans* strain CGMCC6845 is a halotolerant hydrocarbon-degrading bacterium isolated from petroleum-contaminated saline soil. Here we present the 5.0-Mb draft genome sequence of this strain, which will improve our understanding of the diversity of *G. alkanivorans* and the mechanisms of microbial hydrocarbon degradation in saline environment.

## GENOME ANNOUNCEMENT

*Gordonia alkanivorans* can degrade diesel (1), dibenzothiophene, benzothiophene, and other thiophene analogs (2), which may have potential applications in the bioremediation of petroleum-contaminated soils and the biodesulfurization of oil. Therefore, hydrocarbon biodegradation processes (1), biodesulfurization-related genes (3–5), and optimal conditions for the biodesulfurization (6) of *G. alkanivorans* have been well studied. Despite the increasing interest in *G. alkanivorans*, genomic information for this species is still limited. To date, only one strain, *G. alkanivorans* NBRC16433, has been sequenced. Here, the draft genome sequence of the halotolerant hydrocarbon-degrading strain *G. alkanivorans* CGMCC6845 is presented, which was isolated from petroleum-contaminated saline soil in China (7).

Genomic DNA was extracted from a 3-day culture growing in tryptic soy broth and was sequenced using the Illumina HiSeq 2000 platform with a whole-genome shotgun (WGS) strategy. The sequencing produced 8,467,694 paired-end reads with an insert size of 300 bp, yielding about 170-fold coverage. Filtered reads were assembled, scaffolded, gap filled, and validated using SOAPdenovo v2.04 (8), SSPACE v2.0 (9), GapFiller v1.10 (10), and BWA v0.7.4 (11). Final assembly consisted of 74 contigs with an *N*_50_ length of 217,608 bp, which were assembled into 61 scaffolds with an *N*_50_ length of 269,646 bp. Genome annotation was performed using the NCBI Prokaryotic Genome Automatic Annotation Pipeline (PGAAP) (http://www.ncbi.nlm.nih.gov/genome/annotation_prok/).

The genome consists of 5.0 Mb, with a G+C content of 67.5%. A total of 4,476 coding sequences (CDS), 42 pseudogenes, 1 noncoding RNA (ncRNA), 49 tRNA genes, and 3 rRNA operons were identified. A plasmid partitioning gene, *parA*, was detected on contig 20, which suggests the occurrence of a plasmid. We detected 349 tandem repeats using Tandem Repeats Finder v4.07 (14). Neither prophage sequences nor clustered regularly interspaced short palindromic repeat (CRISPR) elements were present, as revealed by PHAST (12) and CRISPRFinder (13). IS3 and IS481 families dominate the insertion sequence (IS) elements, as revealed by ISFinder (14). Average nucleotide identity (ANI) analysis (15) revealed that *G. alkanivorans* CGMCC6845 is phylogenetically related to *G. alkanivorans* NBRC16433 (98.0%).

Nine genes were identified as involved in hydrocarbon degradation, including 3 alkanal monooxygenase genes, 2 catechol 1,2-dioxygenase genes, 2 ring-cleavage dioxygenase genes, 2 benzoate 1,2-dioxygenase genes, and 1 dibenzothiophene desulfurization gene. Moreover, 8 genes were identified as involved in compatible solute synthesis and uptake, including 1 ectoine synthase gene, 2 betaine synthase genes, 3 trehalose synthase genes, and 2 glycine/betaine ABC transport genes, which may enhance the tolerance to osmotic stress. Copper-, arsenic-, and tellurium-resistant genes were detected, which may enhance the resistance to heavy metal. Information about the genome sequence of *G. alkanivorans* CGMCC6845 will give a better understanding of the diversity of *G. alkanivorans* and the mechanisms of microbial hydrocarbon degradation in saline environment.

### Nucleotide sequence accession number.

The draft genome sequence of *G. alkanivorans* CGMCC6845 has been deposited in GenBank under the accession number AYXO00000000. The version described in this paper is the first version.
